# Clonal Lineages, Antimicrobial Resistance, and PVL Carriage of *Staphylococcus aureus* Associated to Skin and Soft-Tissue Infections from Ambulatory Patients in Portugal

**DOI:** 10.3390/antibiotics10040345

**Published:** 2021-03-24

**Authors:** Carolina Ferreira, Sofia Santos Costa, Maria Serrano, Ketlyn Oliveira, Graça Trigueiro, Constança Pomba, Isabel Couto

**Affiliations:** 1 Global Health and Tropical Medicine (GHTM), Instituto de Higiene e Medicina Tropical (IHMT), Universidade Nova de Lisboa (UNL), Rua da Junqueira 100, 1349-008 Lisboa, Portugal; carolinaf@ihmt.unl.pt (C.F.); scosta@ihmt.unl.pt (S.S.C.); a21000870@ihmt.unl.pt (M.S.); mmm0012@ihmt.unl.pt (K.O.); 2 Laboratório de Análises Clínicas Dr. Joaquim Chaves, Av. General Norton de Matos, 71 R/C, 1495-148 Algés, Portugal; graca.trigueiro@jcs.pt; 3 CIISA, Centre of Interdisciplinary Research in Animal Health, Faculty of Veterinary Medicine, University of Lisbon, Avenida da Universidade Técnica, 1300-477 Lisboa, Portugal; cpomba@fmv.ulisboa.pt; 4 GeneVet, Laboratório de Diagnóstico Molecular Veterinário, Rua Quinta da Nora Loja 3B, 2790-140 Carnaxide, Portugal

**Keywords:** *Staphylococcus aureus*, skin and soft-tissue infections, antibiotic resistance, clonal lineages, plasmids, Panton–Valentine leucocidin

## Abstract

*Staphylococcus aureus* (*S. aureus*) is a leading cause of skin and soft-tissue infections (SSTIs) in the community. In this study, we characterized a collection of 34 *S. aureus* from SSTIs in ambulatory patients in Portugal and analyzed the presence of Panton–Valentine leucocidin (PVL)-encoding genes and antibiotic-resistance profile, which was correlated with genetic determinants, plasmid carriage, and clonal lineage. Nearly half of the isolates (15, 44.1%) were methicillin-resistant *Staphylococcus aureus* (MRSA) and/or multidrug resistant (MDR). We also detected resistance to penicillin (33/34, 97.1%), fluoroquinolones (17/34, 50.0%), macrolides and lincosamides (15/34, 44.1%), aminoglycosides (6/34, 17.6%), and fusidic acid (2/34, 5.9%), associated with several combinations of resistance determinants (*blaZ*, *erm*(A), *erm*(C), *msr*(A), *mph*(C), *aacA-aphD*, *aadD*, *aph*(3′)-*IIIa*, *fusC*), or mutations in target genes (*fusA*, *grlA/gyrA*). The collection presented a high genetic diversity (Simpson’s index of 0.92) with prevalence of clonal lineages CC5, CC22, and CC8, which included the MRSA and also most MDR isolates (CC5 and CC22). PVL-encoding genes were found in seven isolates (20.6%), three methicillin-susceptible *Staphylococcus aureus* (MSSA) (ST152-*agr*I and ST30-*agr*III), and four MRSA (ST8-*agr*I). Plasmid profiling revealed seventeen distinct plasmid profiles. This work highlights the high frequency of antimicrobial resistance and PVL carriage in SSTIs-related *S. aureus* outside of the hospital environment.

## 1. Introduction

*Staphylococcus aureus* (*S. aureus*) is a major human pathogen responsible for a wide range of infections both in hospitals and in the community. It is one of the main causes of severe nosocomial infections such as bacteremia and infective endocarditis and in the community is a frequent cause of skin and soft-tissue infections (SSTIs) [1]. Besides their potential severity, infections caused by *S. aureus* are usually difficult to treat due to the frequent acquisition of antimicrobial resistance determinants. In the last decades, there has been an emergence and dissemination of methicillin-resistant *S. aureus* (MRSA) as well as of multidrug-resistant (MDR) strains [2,3]. Consequently, MRSA are now included in the World Health Organization (WHO) list of high-priority bacteria for development of new drugs [4].

*S. aureus* is the most frequent pathogen associated with SSTIs, which can range from minor or superficial infections such as impetigo to life-threating infections such as necrotizing fasciitis [5]. Topical antibiotics that are often used for the prevention or treatment of milder infections include mupirocin, fusidic acid, neomycin, and bacitracin [6,7]. The use of some of these topical antibiotics is particularly relevant in the community/ambulatory settings, where they may not require medical prescription. Other antibiotics for systemic use, such as clindamycin, trimethoprim-sulfamethoxazole, tetracyclines, and linezolid, are also indicated for treatment of severe forms of SSTIs caused by *S. aureus* [6,8]. The frequency of antibiotic-resistant *S. aureus* isolates associated with SSTIs is rising worldwide [9,10,11,12], particularly to fusidic acid and mupirocin, which is probably linked with the widespread use of these antibiotics [3].

Resistance to antibiotics in *S. aureus* can be mediated by several mechanisms, such as antibiotic modification or degradation, target mutation, or antibiotic efflux. Resistance to penicillins can occur by inactivation of the antibiotic molecule through the action of the β-lactamase BlaZ. The *blaZ* gene occurs frequently in *S. aureus* clinical isolates. Resistance to penicillins and other β-lactams, with the exception of fifth-generation cephalosporins, is mediated by the acquisition of the *mecA* gene, which is part of the mobile genetic element SCC*mec* (staphylococcal cassette chromosome *mec*) and encodes for an additional penicillin-binding protein, PBP2a, with low affinity for the β-lactam antibiotics [3]. Resistance to macrolides and lincosamides can occur through several mechanisms, including the acquisition of rRNA methylases-encoding *erm* genes that methylate the binding site of the antibiotics [3]. Resistance to aminoglycosides is associated with acquisition of several genes, like *aacA-aphD* or *aadD* that encode enzymes that modify the antibiotic molecule rendering it inactive [3]. Resistance to fluoroquinolones is usually linked to the occurrence of mutations in the quinolone-resistant determining region (QRDR) of the *grlA/B* and *gyrA/B* genes that encode the DNA topoisomerase IV and DNA gyrase, respectively. Fluoroquinolone resistance can also be conveyed by overexpression of chromosomally-encoded efflux pump genes such as *norA/B/C* and *mepA* [13]. Resistance to fusidic acid can be achieved by the acquisition of the *fusB/C* genes that encode ribosomal protection proteins or by mutations in the *fusA* gene [3].

*S. aureus* produces several virulence factors, including toxins, proteins associated with immune evasion, and tissue-degrading enzymes [1]. The cytotoxin Panton–Valentine leucocidin (PVL), encoded by the genes *lukF-PV* and *lukS-PV* carried on bacteriophage φSa2, is a two-component pore-forming protein that has been strongly associated with *S. aureus* isolates causing skin infections in the community and with necrotizing pneumonia [1]. Nevertheless, the role of PVL in *S. aureus* infection pathogenesis is still not fully elucidated [14]. The *S. aureus* accessory gene regulator *(agr*) locus regulates the expression of several virulence factors like cell-wall-associated and extracellular proteins, contributing to infection severity and persistence. The polymorphism of the *agr* locus allows the classification of *S. aureus* in four predominant *agr* types (I to IV), that may differ in terms of infection type, carriage of virulence factors, and temporal patterns of autoinduction [15].

Most antimicrobial resistance and virulence genes of *S. aureus* are located on mobile genetic elements (MGEs) such as plasmids, bacteriophages, pathogenicity islands, transposons, integrative conjugative elements (ICEs), integrons, and staphylococcal chromosome cassettes (SCCs), which make up to 15–20% of its genome [16]. The acquisition of antimicrobial resistance by *S. aureus* is mostly due to horizontal gene transfer (HGT), and plasmids have been identified as one of the main responsible for the dissemination of resistance genes [17].

Several studies have evaluated the main clones of MRSA circulating both in hospitals and in the community in Portugal, a country with a high prevalence of MRSA [18,19,20,21,22]. However, there have been fewer studies focusing on *S. aureus*, both methicillin-susceptible *Staphylococcus aureus* (MSSA) and MRSA causing SSTIs. The aim of this work was to perform a phenotypic and genotypic characterization of a collection of *S. aureus* isolated from SSTIs in ambulatory patients and to assess their virulence determinants and susceptibility to the main antibiotics used in SSTI therapeutics, correlating their resistance profile to genetic determinants and identifying their main mechanisms of dissemination among *S. aureus* strains.

## 2. Results

### 2.1. Antimicrobial Susceptibility Profile and Correlation with Resistance Determinants

The antimicrobial susceptibility profile of the 34 isolates is described in Table 1. Resistance to penicillin was detected in 97.1% (33/34) of the isolates, and 44.1% (15/34) were MRSA (*mecA*^+^ and cefoxitin resistant). We have also observed resistance to the fluoroquinolones ciprofloxacin and moxifloxacin (50.0%, 17/34), erythromycin (44.1%, 15/34), clindamycin (35.3%, 12/34) either constitutive (2.9%, 1/34) or inducible (32.4%, 11/34), kanamycin (17.6%, 6/34), tobramycin (14.7%, 5/34), amikacin (8.8%, 3/34), gentamycin (2.9%, 1/34), and fusidic acid (5.9%, 2/34). Fifteen isolates (44.1%) were MDR, mainly resistant to β-lactams, fluoroquinolones, macrolides, and lincosamides. All isolates were susceptible to tetracyclines, tigecycline, rifampicin, trimethoprim-sulfamethoxazole, linezolid, chloramphenicol, retapamulin, and quinupristin-dalfopristin. All MRSA isolates were susceptible to ceftaroline. Only one isolate was susceptible to all antibiotics tested. Although no breakpoints or epidemiological cut-off values are established by the European Committee on Antimicrobial Susceptibility Testing (EUCAST) for bacitracin or neomycin (for 30 μg discs), one isolate showed no inhibition zone toward each of these topical antibiotics. The presence of antibiotic-resistance determinants was confirmed for all isolates presenting phenotypic resistance (Table 1). The *blaZ* gene was detected in all isolates resistant to penicillin. All isolates showing resistance to cefoxitin harbored the *mecA* gene. Mutations in QRDR regions of *grlA* and *gyrA* genes were found in different combinations in all the representative fluoroquinolone resistant isolates screened. Resistance to macrolides and lincosamides was associated with *erm*(A), *erm*(C), *msr*(A), and/or *mph*(C). Resistance to aminoglycosides was mainly linked to the *aadD* gene. For the two isolates resistant to fusidic acid, one harbored the *fusC* gene, whereas the other carried three mutations in the *fusA* gene.

### 2.2. Efflux Activity

The presence of increased efflux activity in the 34 isolates was assessed by different approaches. The minimum inhibitory concentrations (MICs) of ethidium bromide (EtBr) for the entire collection ranged from 2 to 16 μg/mL with a unimodal distribution (data not shown). Eleven isolates presented an EtBr MIC of 16 μg/mL, suggesting increased efflux activity in those isolates. In addition, these 11 isolates were also resistant to fluoroquinolones, a class of antibiotics that is substrate of the main efflux pumps in *S. aureus* [13]. To verify the presence of an efflux-mediated resistance in these 11 isolates, the EtBr and ciprofloxacin (CIP) MICs were determined in the presence of the known efflux inhibitors (EIs) thioridazine (TZ) and verapamil (VER) and compared to their original values (Table 2). A significant decrease (four- to eight-fold) in EtBr MICs was observed for all isolates but one, confirming the presence of increased efflux activity in these isolates. However, none of the isolates carried the plasmid-encoded *qacA/B* or *smr* genes, which code for the efflux pumps QacA/B and Smr, respectively, responsible for the extrusion of EtBr and several biocides. These results indicate that the increased efflux activity present in these isolates may be driven by chromosomally-encoded efflux pumps, like NorA, which extrudes EtBr and biocides but also several fluoroquinolones like ciprofloxacin and norfloxacin [13]. The effect of EIs on CIP MICs was less significant, with MIC reductions of two-fold for the majority of the isolates. This result does not exclude the presence of increased efflux activity associated with fluoroquinolone resistance, since these isolates harbor mutations in the QRDR of *grlA* and *gyrA* genes, which are responsible for conferring high-level fluoroquinolone resistance and thus may be hindering the screening of efflux activity associated with resistance to these antibiotics [23].

### 2.3. Main Clonal Lineages and Genetic Diversity of the S. aureus Isolates

**Figure 1:**
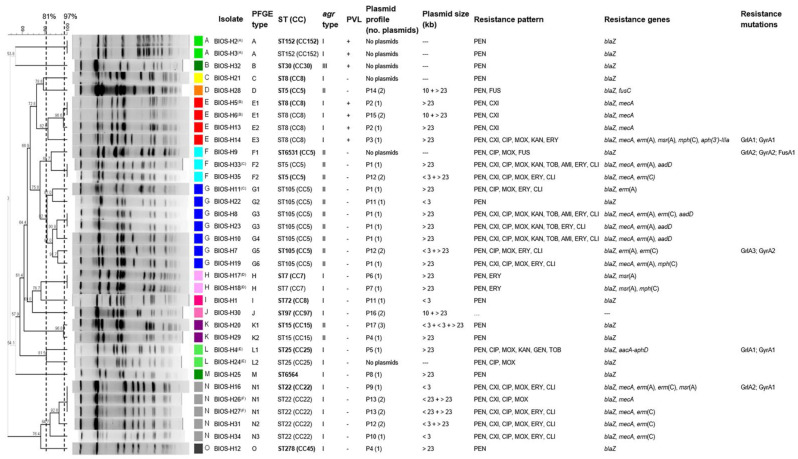
*Sma*I-PFGE macrorestriction profile analysis of the *S. aureus* isolates associated with SSTIs in ambulatory patients and corresponding clonal lineages as determined by MLST and their correlation with PVL carriage and *agr* types, plasmid profiles, and phenotypic and genotypic resistance traits. The pairs of isolates recovered from different anatomical sites of the same patient are marked by (A) to (F), where each letter corresponds to a different patient. The dendrogram was built using Bionumerics and the UPGMA algorithm, using Dice coefficient, and an optimization of 0.5% and tolerance of band of 1%. The dashed lines correspond to the similarity criteria for considering isolates belonging to the same PFGE type (≥81%) or subtype (≥97%). Isolates sharing the same PFGE type or subtype were considered as belonging to the same sequence type (ST). The isolates subjected to MLST are indicated in bold-type. Each plasmid profile corresponds to a unique pattern of undigested and/or *Eco*RI-digested plasmids. CC: clonal complex; ST: sequence type; PFGE: pulsed-field gel electrophoresis; PVL: Panton–Valentine leucocidin; PEN: penicillin; CXI: cefoxitin; ERY: erythromycin; CLI: clindamycin; CIP: ciprofloxacin; MOX: moxifloxacin; KAN: kanamycin; GEN: gentamycin; TOB: tobramycin; AMI: amikacin; and FUS: fusidic acid. Resistance mutations: GrlA1: S80Y; GrlA2: S80F; GrlA3: S80Y, E84G; GyrA1: S84L; GyrA2: E88K; FusA1: A71V, H547Q, G476C.

Analysis of *Sma*I-macrorestriction profiles revealed the presence of 15 pulsed-field gel electrophoresis (PFGE) types (A to O) and 18 subtypes (Figure 1) among the collection studied. The three most common profiles, PFGE types G, N, and E, are represented by seven, five, and four isolates, respectively. An isolate representative of each PFGE type was selected for typing by multilocus sequence typing (MLST). Fourteen sequence types (STs) were identified belonging to 10 clonal complexes. The clonal complexes identified were CC5 (ST5, ST105, and the newly identified ST6531, which is a single-locus variant (SLV) of ST5), CC8 (ST8, ST72), CC152 (ST152), CC30 (ST30), CC7 (ST7), CC97 (ST97), CC15 (ST15), CC25 (ST25), CC22 (ST22), and CC45 (ST278). We also detected a newly identified singleton, ST6564. In general, each ST identified was associated with a single PFGE type, except for ST5 (CC5), associated with PFGE types F and D and ST8 (CC8) associated with PFGE types C and E. The most common PFGE types were linked to ST105 (CC5), ST22 (CC22), and ST8 (CC8). The Simpson’s index of diversity (SID), calculated based upon the PFGE *Sma*I-macrorestriction profiles, revealed a highly diverse *S. aureus* population (SID = 0.92, CI: 0.87–0.98).

### 2.4. Correlation of Strain Lineage with agr Typing and PVL Carriage

The *agr* typing of the *S. aureus* isolates identified *agr*I as the predominant type, which was detected in 20 out of the 34 isolates (58.8%), followed by *agr* type II, identified in 13/34 (38.2%) isolates, and *agr* type III, observed in a single isolate (1/34, 2.9%). No isolate of *agr* type IV was identified. The PVL-encoding genes *lukS-lukF* were detected in seven isolates (20.6%), corresponding to three MSSA (3/19, 15.8%) and four MRSA (4/15, 26.7%), all classified as *agr* type I or III.

As shown in Figure 1, an association was observed between *S. aureus* clonal lineages ST8 (CC8), ST25 (CC25), ST22 (CC22), ST7 (CC7), ST278 (CC45), ST97 (CC97) and ST152 (CC152) and *agr* type I, whereas clonal complexes CC5 and CC15 were linked to *agr* type II, and the singe isolate harboring *agr* type III belonged to ST30 (CC30). The newly identified singleton ST6564 belongs to *agr* type I.

Carriage of PVL was associated with MRSA belonging to ST8 (CC8) and MSSA assigned to ST152 (CC152) or ST30 (CC30).

### 2.5. Correlation of Strain Lineage with Antimicrobial Resistance and Plasmid Profiles

**Figure 2:**
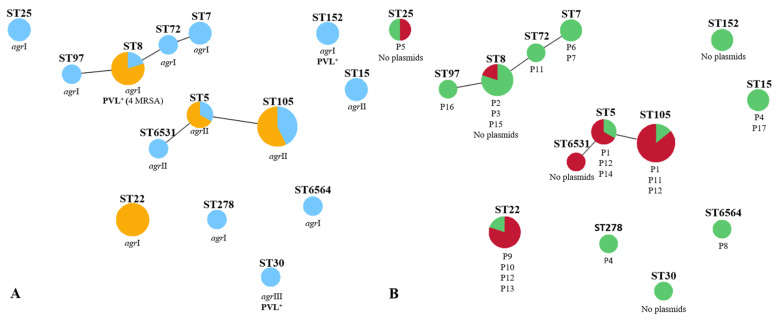
Relation of clonal lineages identified amongst the *S. aureus* associated with SSTIs in ambulatory patients determined using PHYLOViZ software and correlation with (**A**) methicillin resistance status, PVL carriage, and *agr* type; and (**B**) MDR phenotypes and plasmid profile. In panel (**A**), MRSA isolates are displayed in orange whereas MSSA isolates are shown in light blue. In panel (**B**), MDR isolates are presented in red, while non-MDR isolates are shown in green.

Analysis of the methicillin resistance status and clonal lineage showed that the MRSA isolates identified in the collection were restricted to the clonal complexes CC22, CC8 (ST8), and CC5 (ST5 and ST105) (Figure 2). Most isolates from ST22 and clonal lineages of the CC5 presented MDR phenotypes (Figure 2).

The majority of the *S. aureus* isolates studied carried plasmids (28/34, 82.4%), with 19 isolates (55.9%) carrying one plasmid, eight isolates (23.5%) carrying two plasmids, and only one isolate (2.9%) carrying three plasmids. Large plasmids (≥23 kb) were present in most isolates (24/34, 70.6%), alone or in combination with medium or smaller plasmids (10 kb or ≤3 kb). Isolates with large plasmids harbor, in general, a higher number of resistance determinants than those carrying small or no plasmids (Figure 1).

Seventeen plasmid profiles were identified, designated P1 to P17 (Figure 1). For strains carrying a single plasmid, these profiles were defined after restriction with *Eco*RI–profiles P1 to P10. The most frequent profile, P1, is represented by a single large plasmid (>23 kb), identified in six isolates, five of which belonging to ST105 (CC5). All isolates with this plasmid profile are MDR and carry several resistance genes. The second most frequent profile, P12, is shared by three isolates belonging to CC5 and CC22. Isolates of the same clonal complex show a high variety of plasmid profiles. For example, isolates of CC5, CC8, and CC22 have four different plasmids profiles each (Figure 1).

Of the six pairs of isolates recovered from two anatomical sites of the same patient, only one pair was assigned to two distinct PFGE types. Four pairs of isolates were indistinguishable by PFGE, while the remaining pair included subtypes of the same PFGE type. However, different phenotypical or genotypical trait(s) were observed within each pair except one (Figure 1). Isolates of three pairs differed in plasmid content, while two pairs of isolates differed in terms of resistance profile and/or resistance determinants. Another pair of isolates displayed different resistance profile and determinants although sharing the same plasmid profile.

## 3. Discussion

*S. aureus* is a leading cause of bacterial infections not only in healthcare settings but also in the community, many of which are caused by MRSA and MDR strains [2]. According to the most recent data of EARS-Net (European Antimicrobial Resistance Surveillance Network), in 2019, the prevalence of MRSA in bloodstream infections in Portugal was 34.8%. Even though this value has been decreasing over the last decade, it was still the fifth highest registered in Europe [24].

A high frequency of antibiotic resistance was observed in this collection. All isolates except one were resistant to at least one class of antibiotics, mainly β-lactams, and nearly half (44.1%) were MDR, which was unexpected considering they were not from hospitalized patients. However, these isolates were collected from ambulatory patients who could have been under antibiotic therapy or could have had recent contact with hospitals and that might explain the high rates of resistance observed. The 44.1% rate of MRSA identified is higher than the MRSA rates reported in the community (21.6%) in Portugal [19] and in children affected by SSTIs attending a pediatric emergency in Lisbon area (8.6%) [25] in years close to the year of collection of these isolates and is closer to the values observed in hospitals (47.4%) for 2014 [26]. On the other hand, the fact that these patients used laboratory services suggests that these may reflect more complex infections, which may explain the high frequencies of resistance observed [27]. Of the 15 MRSA, 11 (73.3%) were also MDR and the most common pattern was resistance to β-lactams, fluoroquinolones, macrolides, and lincosamides, which is a profile frequently observed in hospital-acquired MRSA (HA-MRSA) [19,20]. Previous studies have shown that there is a high prevalence of HA-MRSA strains in the community in our country, due to dissemination of these strains from the hospital [19,20,21]. The molecular analysis revealed that most of the isolates studied (16/34, 47.1%) presented genetic backgrounds related to hospital-associated lineages, such as CC5 and CC22, which were the predominant HA-MRSA lineages in Portugal during this period, identified in nosocomial or community isolates [18,19]. This finding, together with the use of community laboratory services to treat possible resilient and complex infections, may explain the high frequency of resistance observed in this collection. Regarding MSSA strains, only four strains showed an MDR profile (4/19, 21%). This observed rate of MDR strains is higher than previously reported for other MSSA collected from the community in Portugal [25,28].

With the exception of ST278 and the two new STs, all the other strain lineages identified in this work have been found in other studies in Portugal, with ST8 being the most frequent CA-MRSA clone, while ST30 and ST72 were the most prevalent MSSA clones [18,19,29]. ST278 belongs to CC45 and has been reported in the USA as a MSSA clone [30,31]. Strains of CC45 are prevalent in Portugal [18,19,29], but as far as we know, ST278 has not been yet reported in our country. We have also identified two new STs, ST6531, a SLV of ST5, and the singleton ST6564.

Although the *S. aureus* studied were isolated from patients with SSTIs, a low frequency of resistance was observed toward topical antibiotics, particularly to neomycin and fusidic acid, which are some of the most commonly used for the treatment of SSTIs in the community [7]. Only two isolates (5.9%) were resistant to fusidic acid, and only one isolate (2.9%) did not show inhibition zone to neomycin or bacitracin. The current rates of resistance to fusidic acid reported in the literature for SSTIs-associated *S. aureus* vary geographically, ranging from over 30% in Africa [32] to much lower rates, 2 to 6% in Asia or South America [33,34]. These low levels of resistance to fusidic acid are similar to the ones detected in other contemporary studies in Portugal [19]. The rates of resistance to neomycin and bacitracin in our collection are lower than the ones reported for other CA-MRSA from SSTIs [34]. However, a higher frequency of resistance was detected toward clindamycin (35.3%), an antibiotic also recommended for topical treatment of these infections, in comparison with other CA-MRSA from SSTIs [35,36].

Antibiotic-resistance determinants were identified in all isolates presenting phenotypic resistance (Table 1). The distribution of the fusidic-acid-resistance determinants in *S. aureus* reported in the literature is variable. While some studies report that *fusB* and *fusC* are the most prevalent genes [37], others report *fusA* mutations as the most common mechanism of fusidic-acid resistance [38]. In this study, only two isolates were resistant to fusidic acid. One of these had three mutations in the *fusA* gene, two of which (A71V and H457Q) already associated with resistance to this antibiotic [39,40,41], while the third mutation found, G476C, was described for the first time in this work and could also be contributing to fusidic-acid resistance. The other isolate resistant to fusidic acid carried the *fusC* gene. This is an MSSA that belongs to ST5. Several studies have shown that *fusC* gene can be located in SSC*mec* cassettes, with or without *mecA* gene [42,43].

Screening for mutations in fluoroquinolone-resistant representative isolates identified several patterns of mutations in the QRDR regions of GrlA and GyrA (namely, GrlA S80Y, GyrA S84L; GrlA S80F, GyrA E88K; GrlA S80Y E84G, GyrA E88K; and GrlA S80F GyrA S84L) already associated with high level resistance to these antibiotics [44,45]. These patterns of QRDR mutations were also detected in an earlier study from *S. aureus* clinical isolates in Lisbon [46]. The GrlA S80F and GyrA S84L mutations are the most commonly described in the literature [47,48,49,50,51] and are characteristic of ST22 and some ST8 lineages [51]. In our study, only one isolate carried both mutations and belonged to ST22. The GyrA S84L mutation was also found in one isolate of ST8. The GrlA E84G and S80Y and GyrA E88K mutations are also described in some studies [50,51] but appear to be less frequent. Besides these mutations, the activity of chromosomally-encoded MDR efflux pumps might also be contributing to fluoroquinolone resistance. A subset of fluoroquinolone-resistant isolates presented increased efflux activity of EtBr, a common substrate of MDR efflux pumps like NorA/B/C and MepA, which also extrude fluoroquinolones. No significant reduction in CIP MICs was observed in the presence of EIs, yet the effect of these compounds may be potentially hindered by the presence of QRDR mutations. The absence in this collection of the plasmid-encoded efflux pump genes *qacA/B* or *smr* indicates that the higher EtBr efflux activity detected is probably due to the overexpression of chromosomal efflux pump genes such as *norA/B/C* or *mepA* [13,46]. In the future this, hypothesis can be confirmed be quantifying the expression levels of these genes by RT-qPCR.

Plasmid profiling revealed a high proportion of plasmid-bearing isolates (82.4%) and a high diversity of plasmids, with 17 different profiles identified distributed amongst 14 clonal lineages. Most isolates carried a large plasmid, potentially associated with determinants for resistance to β-lactams, macrolides, lincosamides, and aminoglycosides (Figure 1). These results are similar to the ones found in a previous study that analyzed the plasmid content of a collection of 53 *S. aureus* isolated from a hospital in Lisbon between 2006 and 2007 [52]. The proportion of plasmid-bearing isolates in that study was 83%, and most isolates carried a large plasmid that was frequently associated with resistance to β-lactams, macrolides, and lincosamides. Other studies have also demonstrated that large plasmids are quite common in *S. aureus* and that they can carry several resistance determinants associated with resistance to the classes of antibiotics mentioned above [16].

The occurrence of PVL is linked to the bacteriophage φSa2 and generally associated with community-acquired MRSA (CA-MRSA), being traditionally considered a marker for the identification of CA-MRSA isolates [53], although some CA-MRSA strains do not produce this toxin. Its prevalence in HA-MRSA isolates, albeit lower, has been documented in several countries [54]. PVL is also strongly linked with *S. aureus* isolates collected from skin infections [53,54]. The overall rate of 20.6% of PVL-positive isolates in our set of *S. aureus* associated with SSTIs is lower than the ones reported from children with SSTI attending a pediatric emergency (37%) [25] but higher than the ones reported for other MSSA, CA-MRSA, or HA-MRSA collections in Portugal [19,22,55,56], albeit most of these other collections are not exclusively associated with skin infections. PVL carriage in our set of MRSA isolates appears restricted to the ST8-*agr*I clonal lineage, as found in a previous study by Tavares and colleagues [19]. Interestingly, the single MSSA ST8 isolate of our collection did not harbor PVL. The PVL-positive MSSA detected in our collection belong to the genetic backgrounds ST30-*agr*III and ST152-*agr*I, different from the ones reported in that earlier study [19]. However, PVL-positive ST30 isolates were also detected in children with SSTIs attending a pediatric emergency [25]. The ST8 and ST30 clonal lineages were frequently encountered in isolates from the community and less frequently associated with nosocomial isolates [19,20].

In this study, we performed a phenotypic and genotypic characterization of a collection of *S. aureus* isolated from SSTIs in ambulatory patients. Although this can be considered a relatively small sample, this is a convenience collection that represents the diversity of the population affected by SSTIs in an ambulatory setting over a five-months period where the only condition criteria for inclusion of the *S. aureus* isolates was to be SSTI-related. The genetic diversity of this collection was demonstrated by the high value of the Simpson’s index (SID of 0.92).

## 4. Materials and Methods

### 4.1. Bacterial Isolates

The study comprised a collection of 34 *S. aureus* isolates associated with SSTIs of 28 ambulatory patients. Of the 34 isolates, 31 were collected from wounds (legs, *n* = 17; foot, *n* = 5; armpit, *n* = 3; ear, *n* = 1; and from unidentified sites, *n* = 4), and three were collected from ulcers. Six pairs of isolates (*n* = 12) were collected from different anatomical sites (right/left leg, *n* = 6; right/left armpit, *n* = 2; ear/leg, *n* = 2; and unidentified sites, *n* = 2) of six patients. The isolates were collected between February and June of 2014 at a community clinical diagnostic laboratory in Lisbon, Portugal. All isolates were grown in tryptic soy broth (TSB) (Oxoid™, Hampshire, UK), with shaking or tryptic soy agar (TSA) (Oxoid™) at 37 °C. Species identification was confirmed by amplification of the *nuc* gene following the protocol described by Poulsen and colleagues [57], using the primers described in Table S1 of Supplementary Data.

### 4.2. Antimicrobial Susceptibility Testing

Antimicrobial susceptibility was determined for a panel of 24 antibiotics by disk diffusion in Mueller-Hinton agar (MHA, Oxoid™), according to the EUCAST guidelines [58]. Antibiotics discs were obtained from Oxoid™. The following antibiotic discs (antibiotic content per disc) were used: penicillin (PEN, 1 U), oxacillin (OXA, 1 μg), cefoxitin (CXI, 30 μg), ceftaroline (CPT, 5 μg), ciprofloxacin (CIP, 5 μg), moxifloxacin (MOX, 5 μg), gentamicin (GEN, 10 μg), kanamycin (KAN, 30 μg), tobramycin (TOB, 10 μg), neomycin (NEO, 30 μg), amikacin (AMI, 30 μg), tetracycline (TET, 30 μg), minocycline (MIN, 30 μg), tigecycline (TIG, 15 μg), chloramphenicol (CHL, 30 μg), erythromycin (ERY, 15 μg), clindamycin (CLI, 2 μg), quinupristin/dalfopristin (QD, 15 μg), linezolid (LIN, 10 μg), trimethoprim-sulfamethoxazole (TRS, 25 μg), rifampicin (RIF, 5 μg), bacitracin (BAC, 10 U), fusidic acid (FUS, 10 μg), and mupirocin (MUP, 200 μg). The D-zone test was performed for detection of inducible clindamycin resistance, and the penicillin inhibition zone was examined to detect production of β-lactamases. Susceptibility testing to ceftaroline was performed for MRSA isolates only. Susceptibility to retapamulin (RET) was evaluated by determination of MICs by the two-fold microdilution method with cation-adjusted Mueller-Hinton broth (CAMHB, Oxoid™), according to the Clinical and Laboratory Standards Institute (CLSI) guidelines [59]. Retapamulin was acquired in powder form from Sigma-Aldrich (St. Louis, MO, USA), dissolved in dimethyl sulfoxide, and diluted in water with 10% β-cyclodextrin [60]. The reference strain *S. aureus* ATCC^®^29213™ was used as quality control. Isolates resistant to one antibiotic of at least three classes of antibiotics were considered multidrug resistant [61].

### 4.3. Detection of Resistance Genes by PCR

Total DNA was extracted from each isolate by the boiling method as described by Alexopoulou and colleagues [62]. All isolates were screened by PCR for the presence of the resistance genes *mecA* and *blaZ* and plasmid-encoded efflux pump genes *qacA*/*B* and *smr* (reduced susceptibility to biocides and EtBr). Isolates presenting phenotypic resistance to antibiotics were also screened for the presence of the genes *erm*(A), *erm*(B), *erm*(C), *msr*(A), *mph*(C), *vga*(A), *vga*(C) (resistance to macrolides, lincosamides, and streptogramins), *aadD*, *aph*(3′)-*IIIa*, *aacA-aphD* (resistance to aminoglycosides), *fusB*, and *fusC* (resistance to fusidic acid) using the primers described in Table S1 of Supplementary Data.

### 4.4. Screening of Mutations in grlA, gyrA, and fusA Genes

Mutations in the QRDRs of *grlA* and *gyrA* genes associated with fluoroquinolone resistance were screened for representative isolates, chosen according to their PFGE types. Mutations in the *fusA* gene were screened for isolates presenting resistance to fusidic acid. The primers used for amplification and sequencing of *grlA*, *gyrA*, and *fusA* genes are described in Table S1. Amplification products were purified using the kit NZYGelpure (NZYTech, Lisboa, Portugal) and sequenced. Sequences were analyzed using the programs SnapGene Viewer (GSL Biotech; available at snapgene.com) and blastx (NCBI, Bethesda, MD, USA).

### 4.5. Evaluation of Efflux Activity

The presence of increased efflux activity was evaluated by (i) determining the EtBr MIC [63] and (ii) determination of EtBr and CIP MICs in the presence of the EIs TZ and VER [23,46]. MICs of EtBr, CIP, TZ, and VER (Sigma-Aldrich) were determined by the two-fold broth microdilution method. Briefly, from overnight cultures, a cellular suspension equivalent to McFarland 0.5 was prepared in CAMHB and aliquoted in 96-well plates containing two-fold dilutions of the compound to be tested. Plates were incubated at 37 °C for 18 h, and the MIC registered as the lowest concentration of compound that inhibited visible growth. EtBr and CIP MICs were then redetermined in the presence of TZ and VER at 12.5 μg/mL and 400 μg/mL, respectively, corresponding to a subinhibitory concentration (1/2 MIC) [23]. The 96-well plates were prepared as described previously, except for the addition of a 0.01 mL aliquot of TZ or VER to each well prior to inoculation of the plate. Each assay was performed in duplicate. A four-fold, or higher, decrease in MICs values in the presence of EIs is indicative of inhibition of efflux activity [23].

### 4.6. Plasmid DNA Extraction and Profiling

Plasmid DNA of each isolate was extracted with the kit NZYMiniprep (NZYTech), adding 35 μg/mL of lysostaphin (Sigma-Aldrich) in the cell lysis step with buffer A1, followed by an incubation at 37 °C for 90 min. For isolates carrying a single plasmid, plasmid DNA was digested with 10 U of the enzyme *Eco*RI (NZYTech). The reaction mixture was incubated at 37 °C for 90 min and inactivated at 65 °C for 20 min. Restriction profiles were analyzed by 1% (*w*/*v*) agarose gel electrophoresis for 90 min.

### 4.7. Detection of lukSF Genes

The presence of the determinants *lukF-PV* and *lukS-PV* encoding PVL was screened by PCR, using the primers described in Table S1 of the Supplementary Data.

### 4.8. Molecular Typing

All isolates were characterized by PFGE. *Sma*I-PFGE was performed as previously described [64], and macrorestriction profiles were analyzed with the Bionumerics software v 7.6 using the Dice coefficient and dendrograms built based on the UPGMA algorithm, considering a band tolerance of 1% and an optimization of 0.5%. Isolates presenting macrorestriction profiles with a similarity ≥81% or ≥97% were considered as belonging to the same PFGE type or subtype, respectively [65]. The genetic diversity of the collection was calculated, based on PFGE types, by Simpson’s index of diversity with a confidence interval of 95% [66].

A subset of isolates representative of each PFGE type was further analyzed by MLST. Isolates sharing the same PFGE type or subtype were considered as belonging to the same ST. Internal fragments of the seven housekeeping genes *arcC*, *aroE*, *glpF*, *gmk*, *pta*, *tpi*, and *yqiL* were amplified by PCR and sequenced using the primers and conditions previously described [67,68]. Allelic profiles and STs were obtained from MLST database (PubMLST.org (accessed on 28 December 2020). New alleles and ST profiles were submitted to PubMLST for validation and allele/ST assignment. The relationship between clonal lineages were inferred with the PHYLOViZ freeware using the goeBurst algorithm [69].

*agr* typing of all isolates was performed according to the protocol described by Lina and colleagues [70]. The set or primers used for *agr* typing is described in Table S1.

## 5. Conclusions

This work demonstrates a high prevalence of antibiotic resistance in *S. aureus* of SSTIs from outside the hospital environment, correlating it with the presence of several antibiotic-resistance determinants and a high prevalence of PVL-positive isolates, assigned to three MSSA (ST152-*agr*I and ST30-*agr*III) and four MRSA (ST8-*agr*I) isolates. This study also highlights the phenotypic and genotypic variability that may be present in *S. aureus* isolates causing infection in distinct anatomical sites of the same patient. The high diversity of plasmids identified in this collection demonstrates the important role these MGEs have in the transmission of antimicrobial resistance in *S. aureus* and the relevance of studying these elements to further prevent the dissemination of MDR strains.

## Figures and Tables

**Table 1:** Antimicrobial resistance phenotypes of the 34 *S. aureus* included in this study and correlation with resistance determinants.

Class	Antibiotic	Resistant Isolates (%)	Resistance Determinants (No. Isolates); [Mutations]
β-lactams	PEN	33 (97.1%)	*blaZ* (33) *mecA* (15)
	CXI	15 (44.1%)	
Fluoroquinolones	CIP	17 (50%)	Mutations in GrlA [S80Y, E84G, S80F] and GyrA [S84L, E88K]
	MOX	17 (50%)	
Macrolides/Lincosamides	ERY	15 (44.1%)	*erm*(A) (9), *erm*(C) (7) *msr*(A) (4), *mph*(C) (3)
	CLI	12 (35.3%)	
Aminoglycosides	KAN	6 (17.6%)	*aadD* (4), *aacA-aphD* (1) *aph(3′)-IIIa* (1)
	TOB	5 (14.7%)	
	AMI	3 (8.8%)	
	GEN	1 (2.9%)	
Fusidanes	FUS	2 (5.9%)	*fusC* (1) FusA mutations [A71V, H457Q, G476C]

PEN: penicillin; CXI: cefoxitin; ERY: erythromycin; CLI: clindamycin; CIP: ciprofloxacin; MOX: moxifloxacin; KAN: kanamycin; GEN: gentamycin; TOB: tobramycin; AMI: amikacin; and FUS: fusidic acid.

**Table 2:** The effect of the efflux inhibitors thioridazine and verapamil on ethidium bromide and ciprofloxacin MICs for selected *S. aureus* isolates.

Isolate	MIC (μg/mL)					
	EtBr	EtBr + TZ	EtBr + VER	CIP	CIP + TZ	CIP + VER
BIOS-H4	16	**4**	**2**	16	16	16
BIOS-H7	16	8	8	512	256	256
BIOS-H8	16	8	**4**	512	256	256
BIOS-H10	16	8	**4**	512	256	256
BIOS-H11	16	**4**	**2**	256	128	128
BIOS-H14	16	**4**	**2**	128	64	64
BIOS-H19	16	**4**	**2**	512	256	256
BIOS-H23	16	**4**	**4**	256	128	256
BIOS-H24	16	**2**	**2**	32	16	16
BIOS-H31	16	**4**	**2**	512	**128**	**128**
BIOS-H33	16	**4**	**2**	512	256	256

MIC: minimum inhibitory concentration; EtBr: ethidium bromide; CIP: ciprofloxacin; TZ: thioridazine; VER: verapamil. Bold-type numbers indicate MIC reductions ≥ four-fold in the presence of EIs when compared to the original MIC values.

## Data Availability

All relevant data have been provided in the paper. Raw data can be provided by the authors upon reasonable request.

## Supplementary Materials

The following is available online at https://www.mdpi.com/2079-6382/10/4/345/s1, Table S1: Primers used in this study.
