# Supplementary material for: Clonal Lineages, Antimicrobial Resistance, and PVL Carriage of Staphylococcus aureus Associated to Skin and Soft-Tissue Infections from Ambulatory Patients in Portugal
Source: Antibiotics (Basel). 2021 Mar 24;10(4):345. doi: 10.3390/antibiotics10040345 (PMC8063795; doi:10.3390/antibiotics10040345)
Supplement: Supplementary file 1 [file antibiotics-10-00345-s001.pdf]

antibiotics

MDPI

Article

Clonal Lineages, Antimicrobial Resistance, and PVL Carriage of *Staphylococcus aureus* Associated to Skin and Soft-Tissue Infections from Ambulatory Patients in Portugal

Carolina Ferreira [1], Sofia Santos Costa [1], Maria Serrano [1], Ketlyn Oliveira [1], Graça Trigueiro [2], Constança Pomba [3,4], and Isabel Couto [1,*]

[1] Global Health and Tropical Medicine, GHTM, Instituto de Higiene e Medicina Tropical, IHMT, Universidade Nova de Lisboa, UNL, Rua da Junqueira 100, 1349-008 Lisboa, Portugal; carolinaf@ihmt.unl.pt (C.F.); scosta@ihmt.unl.pt (S.S.C.); a21000870@ihmt.unl.pt (M.S.); mmm0012@ihmt.unl.pt (K.O.)

[2] Laboratório de Análises Clínicas Dr. Joaquim Chaves, Av. General Norton de Matos, 71 R/C, 1495-148 Algés, Portugal; graca.trigueiro@jcs.pt

[3] CIISA, Centre of Interdisciplinary Research in Animal Health, Faculty of Veterinary Medicine, University of Lisbon, Avenida da Universidade Técnica, 1300-477 Lisboa, Portugal; cpomba@fmv.ulisboa.pt

[4] GeneVet, Laboratório de Diagnóstico Molecular Veterinário, Rua Quinta da Nora Loja 3B, 2790-140 Carnaxide, Portugal

* Correspondence: icouto@ihmt.unl.pt; Tel.: +351-21-3652652; Fax: +351-21-3632105

Table S1. Primers used in this study.

Target Gene	Primers	Nucleotide Sequence (5′-3′)	Amplicon Size (bp)	Reference
		***S. aureus* identification**		
nuc	nuc-1	TCAGCAAATGCATCACAAACAG	255	[1]
	nuc-2	CGTAAATGCACTTGCTTCAGG		
		**MLST**		
arcC	arcC_Fw	TTGATTCACCAGCGCGTATTGTC	456	[2]
	arcC_Rv	AGGTATCTGCTTCAATCAGCG		[3]
aroE	aroE_Fw	ATCGGAAATCCTATTTCACATTC	456	[3]
	aroE_Rv	GGTGTTGTATTAATAACGATATC		
glpF	glpF_Fw	CTAGGAACTGCAATCTTAATCC	465	[3]
	glpF_Rv	TGGTAAAATCGCATGTCCAATTC		
gmk	gmk_Fw	ATCGTTTTATCGGGACCATC	429	[3]
	gmk_Rv	TCATTAACTACAACGTAATCGTA		
pta	pta_Fw	GTTAAAATCGTATTACCTGAAGG	474	[3]
	pta_Rv	GACCCTTTTGTTGAAAAGCTTAA		
tpi	tpi_Fw	TCGTTCATTCTGAACGTCGTGAA	402	[3]
	tpi_Rv	TTTGCACCTTCTAACAATTGTAC		
yqiL	yqiL_Fw	CAGCATACAGGACACCTATTGGC	516	[3]
	yqiL_Rv	CGTTGAGGAATCGATACTGGAAC		
		**Screening of resistance genes and mutations**		
mecA	mecA_Fw	GGTCCCATTAACTCTGAAG	1040	[4]
	mecA_Rv	AGTTCTGCAGTACCGGATTTGC		
blaZ	blaZ_Fw	GATAAGAGATTTGCCTATGC	533	[5]
	blaZ_Rv	GCATATGTTATTGCTTGACC		
***erm*(A)**	erm(A)_Fw	AAGCGGTAAACCCCTCTGAG	442	[6]
	erm(A)_Rv	TCAAAGCCTGTCGGAATTGG		

bp: base pair; **Fw:** "forward"; **Rv:** "reverse".

Table S1. Primers used in this study.

Target Gene	PRIMERS	Nucleotide Sequence (5′-3′)	Amplicon Size (bp)	Reference
***erm*(B)**	erm(B)_Fw	TGGAACAGGTAAAGGGCATT	433	[7]
	erm(B)_Rv	TGTGGTATGGCGGGTAAGTT		
***erm*(C)**	erm(C)_Fw	TCGTAACTGCCATTGAAATA	348	[7]
	erm(C)_Rv	TCACTTTAGGTTTAGGATGAAA		
***msr*(A)**	msr(A)_Fw	GATTGTCCCAAGCCAGTAAA	445	This study
	msr(A)_Rv	GCCATTTGCACTTTAGGAGA		
***mph*(C)**	mph(C)_Fw	ATGACTCGACATAATGAAAT	900	[8]
	mph(C)_Rv	CTACTCTTTCATACCTAACTC		
***vga*(A)**	vga(A)_Fw	ACCCGAGACATCTTCACCAC	400	[9]
	vga(A)_Rv	GGAAATTGACGAGGGGAGA		
***vga*(C)**	vga(C)_Fw	ACGAATAAAGGGATCGAAGC	510	This study
	vga(C)_Rv	AGCACATGCACAGGTTTGTA		
***aac*(A)-*aph*(D)**	aacA-aphD_Fw	CAGAGCCTTGGGAAGATGAAG	348	[10]
	aacA-aphD_Rv	CCTCGTGTAATTCATGTTCTGGC		
aph*(3′)-*IIIa	aph(3′)-IIIa_Fw	CCGCTGCGTAAAAGATAC	609	[11]
	aph(3′)-IIIa_Rv	GTCATACCACTTGTCCGC		
***aad*D**	aadD_Fw	GGAAGCAGAGTTCAGCCATG	266	[12]
	aadD_Rv	TGCCTGCATATTCAAACAGC		
fusB	fusB_Fw	ATTCAATCGGAAACCTATAATGATA	292	[13]
	fusB_Rv	TTATATATTTCCGATTTGATGCAAG		
fusC	fusC_Fw	GATATTGATATCTCGGACTT	128	[14]
	fusC_Rv	AGTTGACTTGATGAAGGTAT		
fusA	fusA_Fw	TTTACCCTGAGTGTGTTCT	2250	[15]
	fusA_Rv	TACATTTAAGCTCACCTTGT		
	FusA_Int_A_F	CGCCAACTCACGTGAAGAAA	1025	
	FusA_Int_B_R	ATTGACCACGACCACCAGAT	1351	
	FusA_Int_C_R	TGCTTCACGTGCTTCTTCAG	639	
***qac*A/B**	qacA/B_Fw	GCTGCATTTATGACAATGTTTG	628	[16]
	qacA/B_Rv	AATCCCACCTACTAAAGCAG		
smr	smr_Fw	ATAAGTACTGAAGTTATTGGAAGT	285	[17]
	smr_Rv	TTCCGAAAATGTTTAACGAAACTA		
gyrA	gyrA_Fw	CCAGTGAAATGCGTGAATC	514	[18]
	gyrA_Rv	TGTGGTGGAATATTCGTTGC		
grlA	grlA_Fw	AGGTGATCGCTTTGGAAGA	501	[18]
	grlA_Rv	TGGTGGTATATCTGTCGCGTA		
**Screening of PVL encoding genes and *agr* typing**				
lukS-lukF	lukSF_Fw	CATTAGGTAAAATGTCTGG	431	This study
	lukSF_Rv	GCATCAACTGTATTGGAT		
agr locus	pan_agr_Fw	ATGCACATGGTGCACATGC		[19]
	agrI_Rv	GTCACAAGTACTATAAGCTGCGAT	439	
	agrII_Rv	TATTACTAATTGAAAAGTGCCATAGC	572	
	agrIII_Rv	GTAATGTAATAGCTTGTATAATAA-TACCCAG	321	
	agrIV_Rv	CGATAATGCCGTAATACCCG	657	

bp: base pair; **Fw:** "forward"; **Rv:** "reverse".

References

1. Poulsen, A.B.; Skov, R.; Pallesen, L.V. Detection of methicillin resistance in coagulase-negative staphylococci and in staphylococci directly from simulated blood cultures using the EVIGENE MRSA Detection Kit. *J Antimicrob Chemother* **2003**, *51*, 419-421. doi: 10.1093/jac/dkg084

2. Crisóstomo, M.I.; Westh, H.; Tomasz, A.; Chung, M.; Oliveira, D.C.; de Lencastre, H. The evolution of methicillin resistance in *Staphylococcus aureus*: similarity of genetic backgrounds in historically early methicillin susceptible and resistant isolates and contemporary epidemic clones. *Proc Natl Acad Sci USA* **2001**, *98*, 9865–9870. doi: 10.1073/pnas.161272898
3. Enright, M.C.; Day, N.P.; Davies, C.E.; Peacock, S.J.; Spratt, B.G. Multilocus sequence typing for characterization of methicillin-resistant and methicillin-susceptible clones of *Staphylococcus aureus*. *J Clin Microbiol* **2000**, *38*, 1008–1015. doi: 10.1128/JCM.38.3.1008-1015.2000
4. Petinaki, E.; Arvaniti, A.; Dimitracopoulos, G.; Spiliopoulou, I. Detection of *mecA*, *mecR1* and *mecI* genes among clinical isolates of methicillin-resistant staphylococci by combined polymerase chain reactions. *J Antimicrob Chemother* **2001**, *47*, 297-304. doi: 10.1093/jac/47.3.297
5. Milheiriço, C.; Portelinha, A.; Krippahl, L.; de Lencastre, H.; Oliveira, D.C. Evidence for a purifying selection acting on the β-lactamase locus in epidemic clones of methicillin-resistant *Staphylococcus aureus*. *BMC Microbiol* **2011,** *11*, 76. doi: 10.1186/1471-2180-11-76
6. Jensen, L.B.; Hammerum, A.M.; Bager, F.; Aarestrup, F.M. Streptogramin resistance among *Enterococcus faecium* isolated from production animals in Denmark in 1997. *Microb Drug Resist* **2002**, *8*, 369-374. doi: 10.1089/10766290260469642
7. Costa, S.S.; Palma, C.; Kladec, K.; Fessler, A.T.; Viveiros, M.; Melo-Cristino, J.; Schwarz, S.; Couto, I. Plasmid-borne antimicrobial resistance of *Staphylococcus aureus* isolated in a hospital in Lisbon, Portugal. *Microb Drug Resist* **2016**, *22*, 617-626. doi: 10.1089/mdr.2015.0352
8. Schnellmann, C.; Gerber, V.; Rossano, A.; Jaquier, V.; Panchaud, Y.; Doherr, M.G.; Thomann, A.; Straub, R.; Perreten, V. Presence of new *mecA* and *mph*(C) variants conferring antibiotic resistance in *Staphylococcus* spp. isolated from the skin of horses before and after clinic admission. *J Clin Microbiol* **2006**, *44*, 4444-4454. doi: 10.1128/JCM.00868-06
9. Couto, N.; Belas, A.; Kadlec, K.; Schwarz, S.; Pomba, C. Clonal diversity, virulence patterns and antimicrobial and biocide susceptibility among human, animal and environmental MRSA in Portugal. *J Antimicrob Chemother* **2015**, *71*, 1479-1487. doi: 10.1093/jac/dkv141
10. Vakulenko, S.B.; Donabedian, S.M.; Voskresenskiy, A.M.; Zervos, M.J.; Lerner, S.A.; Chow, J.W. Multiplex PCR for detection of aminoglycoside resistance genes in enterococci. *Antimicrob Agents Chemother* **2003**, *47*, 1423-1426. doi: 10.1128/aac.47.4.1423-1426.2003
11. Perreten, V.; Vorlet-Fawer, L.; Slickers, P.; Ehricht, R.; Kuhnert, P.; Frey, J. Microarray-based detection of 90 antibiotic resistance genes of Gram-positive bacteria. *J Clin Microbiol* **2005**, *43*, 2291-2302. doi: 10.1128/JCM.43.5.2291-2302.2005
12. Kobayashi, N.; Alam, M.; Nishimoto, Y.; Urasawa, S.; Uehara, N.; Watanabe, N. Distribution of aminoglycoside resistance genes in recent clinical isolates of *Enterococcus faecalis*, *Enterococcus faecium* and *Enterococcus avium*. *Epidemiol Infect* **2001**, *126*, 197-204. doi: 10.1017/s0950268801005271
13. O'Neill, A.J.; Larsen, A.R.; Henriksen, A.S.; Chopra, I. A fusidic acid-resistant epidemic strain of *Staphylococcus aureus* carries the *fusB* determinant, whereas *fusA* mutations are prevalent in other resistant isolates. *Antimicrob Agents Chemother* **2004**, *48*, 3594-3597. doi: 10.1128/AAC.48.9.3594-3597.2004
14. Castanheira, M.; Watters, A.A.; Bell, J.M.; Turnidge, J.; Jones, R.N. Fusidic acid resistance rates and prevalence of resistance mechanisms among *Staphylococcus spp*. isolated in North America and Australia, 2007-2008. *Antimicrob. Agents Chemother* **2010**, *54*, 3614-3617. doi: 10.1128/AAC.01390-09
15. Frosini, S.M.; Bond, R.; Rantala, M.; Grönthal, T.; Rankin, S.C.; O'Shea, K.; Timofte, D.; Schmidt, V.; Lindsay, J.; Loeffler, A. Genetic resistance determinants to fusidic acid and chlorhexidine in variably susceptible staphylococci from dogs. *BMC Microbiol* **2019**, *19*, 81. https://doi.org/10.1186/s12866-019-1449-z
16. Anthonisen, I.L.; Sunde, M.; Steinum, T.M.; Sidhu, M.S.; Sorum, H. Organization of the antiseptic resistance gene *qacA* and Tn552-related β-lactamase genes in multidrug-resistant *Staphylococcus haemolyticus* strains of animal and human origins. *Antimicrob Agents Chemother* **2002**, *46*, 3606-3612. doi: 10.1128/AAC.46.11.3606-3612.2002
17. Bjorland, J.; Sunde, M.; Waage, S. Plasmid-Borne *smr* gene causes resistance to quaternary ammonium compounds in bovine *Staphylococcus aureus*. *J Clin Microbiol* **2001**, *39*, 3999-4004. doi: 10.1128/JCM.39.11.3999-4004.2001
18. Costa, S.S.; Viveiros, M.; Rosato, A.E.; Melo-Cristino, J.; Couto, I. Impact of efflux in the development of multidrug resistance phenotypes in *Staphylococcus aureus*. *BMC Microbiol* **2015**, *15*, 232. doi: 10.1186/s12866-015-0572-8
19. Lina, G.; Boutite, F.; Tristan, A.; Bes, M.; Etienne, J.; Vandenesch, F. Bacterial competition for human nasal cavity colonization: role of Staphylococcal *agr* alleles. *Appl Environ Microbiol* **2003**, *69*, 18-23. doi: 10.1128/aem.69.1.18-23.2003
